# Unilateral Myelinated Retinal Nerve Fiber Layer, High Myopia, and Amblyopia: A Case Report

**DOI:** 10.7759/cureus.21469

**Published:** 2022-01-21

**Authors:** Sara Pereira, Alberto Lemos, Paula Tenedório

**Affiliations:** 1 Ophthalmology, Hospital Pedro Hispano, Porto, PRT

**Keywords:** pediatric ophthalmology and strabismus, myelinated retinal nerve fiber layer, myopia, strabismus, amblyopia

## Abstract

Straatsma syndrome corresponds to the presence of myelinated retinal nerve fibers with ipsilateral myopia and amblyopia. Strabismus is also a common finding in this entity, and patients with strabismus usually present a poor visual prognosis. We report the case of a two-year-old male diagnosed with Straatsma syndrome with a follow-up period of 2.5 years. Despite the early diagnosis and institution of treatment, there was no significant response to therapy. Early recognition of Straatsma syndrome and aggressive treatment of amblyopia may result in better outcomes.

## Introduction

Myelinated retinal nerve fibers appear as white or gray striated patches in the area of distribution of retinal nerve fibers. They are usually asymptomatic and detected incidentally during ocular fundus examination, with an estimated incidence of 1% and bilateral involvement in 7.7% of individuals [1]. In rare cases, myelinated retinal nerve fibers may occur in association with retinal vascular, craniocephalic, and hamartoneoplastic disorders [2]. The presence of myelinated retinal nerve fibers associated with ipsilateral myopia and amblyopia corresponds to Straatsma syndrome [3]. Strabismus is also a common finding. This syndrome is a rare entity, which is usually associated with a poor visual prognosis.

## Case presentation

**Figure 1:**
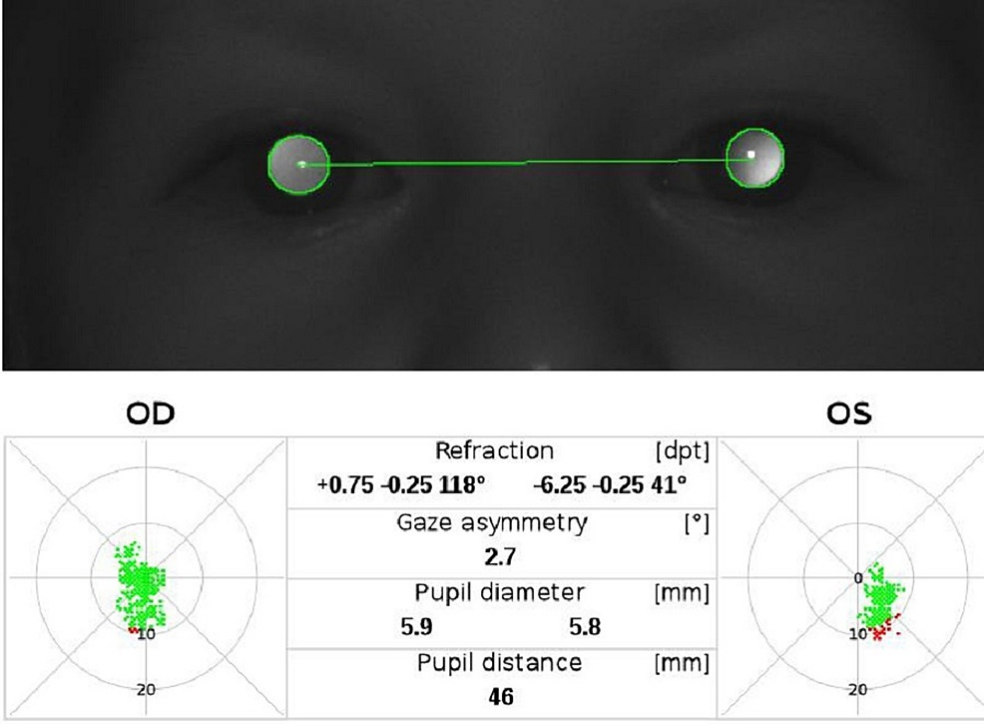
Automated Plusoptix

A two-year-old male presented with anisometropia from high myopia in the left eye. The condition was detected from a visual screening, performed with the automated Plusoptix A09® (PlusoptiX GmbH, Nuremberg, Germany) (Figure 1).

**Figure 2:**
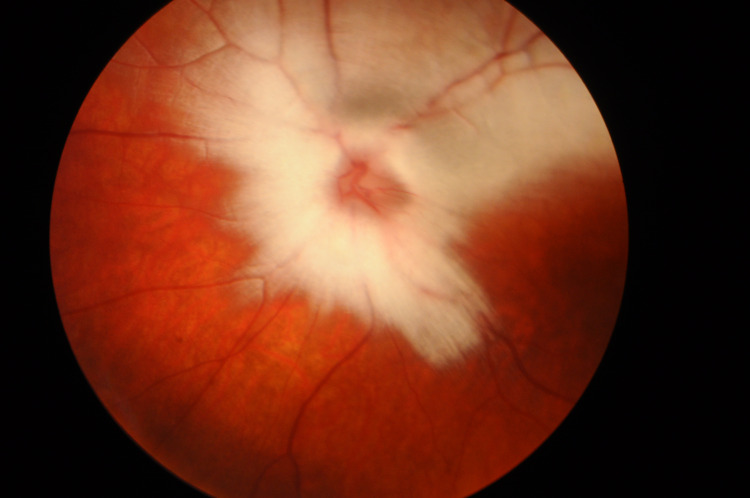
Left eye with dense myelinated nerve fibers

**Figure 3:**
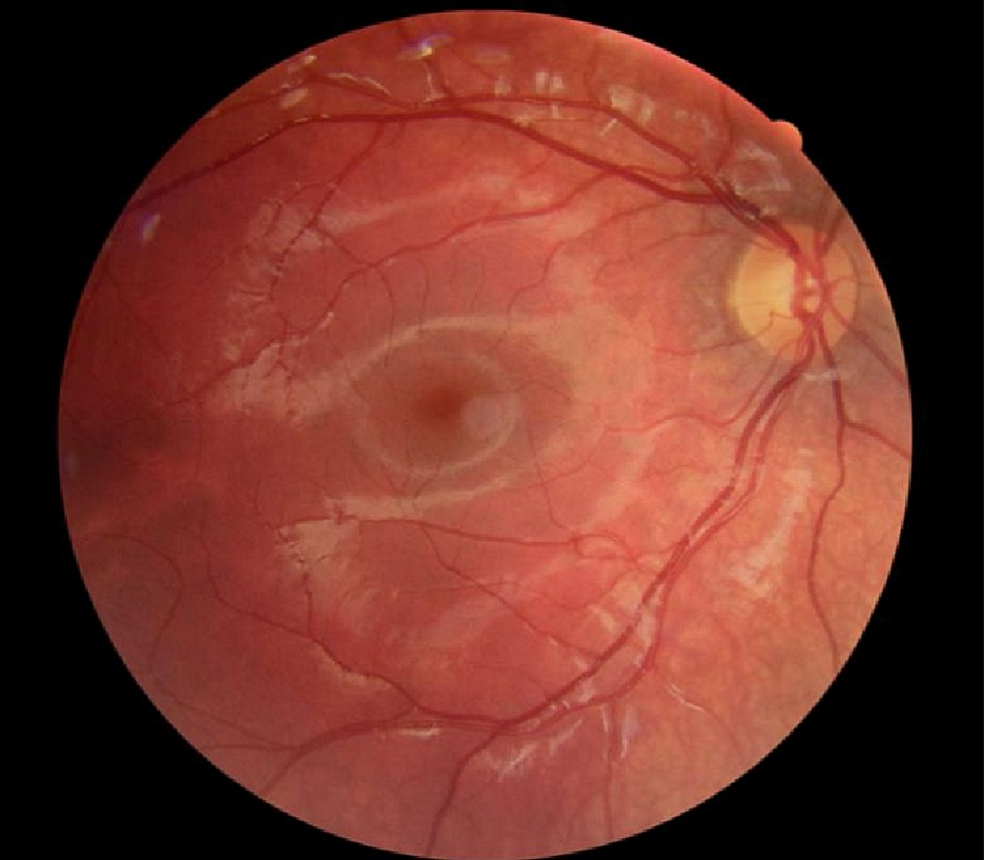
Normal right eye retinography

Cycloplegic retinoscopy revealed a refractive error of a +1.25 sphere in the right eye and a -2.75 diopter sphere with a -2.50 diopter cylinder at 70° in the left eye. Fundus ocular examination showed dense left myelinated retinal nerve fibers around the optic disc and up to the superior vascular arcade and normal right eye fundoscopy (Figures 2, 3).

Glasses with full optical correction were prescribed, and the child was evaluated three months later, presenting a best-corrected visual acuity (BCVA) of 8/10 in the right eye and 1/10 in the left eye. At this point, amblyopia treatment was started with six hours daily of right eye occlusion.

Three months after starting the occlusion therapy, a new cycloplegic retinoscopy revealed an increase in myopia, with a new prescription of -4.00 and -3.00 × 70° to the left eye and a BCVA of 2/10. The mother reported incomplete treatment, accomplishing only two hours of daily occlusion. Given the lack of compliance to therapy, total right eye occlusion was prescribed for one month, but no improvement was noted after that time; therefore, the total time of occlusion was reduced to six hours daily.

In the moment of the last observation, two and a half years later from diagnosis, the patient maintained a BCVA of 2/10 in the left eye (and 10/10 in the right eye), with no development of strabismus. Daily occlusion of the right eye (six hours) was advised during the entire period of follow-up. Regardless of the prescribed occlusion, the parents still reported very irregular compliance. Visual evoked potentials were normal in the right eye and showed moderate dysfunction of the left optical path, compatible with the amblyopia presented.

## Discussion

Patients with Straatsma syndrome present axial myopia instead of refractive myopia [3]. Visual deprivation in early life can induce axial elongation with resultant myopia, and dense myelinated retinal fibers may cause visual deprivation by obscuring vision and producing optical defocus [2,4]. Amblyopia associated with myelinated retinal fibers portends a poor prognosis and a worse response to treatment than anisometropic amblyopia without myelinated retinal fibers [3,5]. The presence of strabismus is frequent in Straatsma syndrome and is also correlated with worse visual outcomes [2], with younger patients without strabismus and with parafoveal fixation reported to respond best to amblyopia treatment [3].

Full optical correction of myopia and treatment of amblyopia should be initiated as soon as possible. Despite the presentation and prognosis, there are some reports with good visual results; therefore, intensive visual rehabilitation should always be attempted [5-7]. The amount of anisometropia and the total area of myelination are important prognostic factors for visual improvement [5].

Our case represents an early diagnosis of Straatsma syndrome identified through a visual screening. In our case, there was no macular involvement and no development of strabismus, which are recognized as good prognostic factors. Despite the initial presentation, the patient showed a refractory response to the treatment of amblyopia, which could be explained by the lack of complete adherence. Amblyopia treatment is long and requires full cooperation from the patient and the family members. We believe that this case could have presented better outcomes with strict adherence to therapy.

## Conclusions

Straatsma syndrome is a rare entity of persistence of myelinated retinal fibers associated with visual dysfunction. Although treatment of amblyopia in these cases is usually linked to poor visual outcomes, implementation of infantile visual screenings can aid in early diagnosis and treatment, improving the visual prognosis. Complete adherence to amblyopia treatment is fundamental for a positive response.
